# Correlations between linear sprint with the ball, linear sprint without the ball, and change-of-direction without the ball in professional female soccer players

**DOI:** 10.1038/s41598-022-27255-y

**Published:** 2023-01-02

**Authors:** Artur Avelino Birk Preissler, Pedro Schons, Filipe Manuel Clemente, Guilherme Droescher de Vargas, Lucas Moraes Klein, Ana Filipa Silva, Hadi Nobari, Luiz Fernando Martins Kruel

**Affiliations:** 1 Faculdade SOGIPA, Porto Alegre, RS Brazil; 2 School of Physical Education, Physiotherapy and Dance, Swimming Center, Federal University of Rio Grande do Sul, Room 18, Felizardo Street, 750, Porto Alegre, RS 90690-200 Brazil; 3 Escola Superior Desporto e Lazer, Instituto Politécnico de Viana do Castelo, Rua Escola Industrial e Comercial de Nun’Álvares, 4900-347 Viana do Castelo, Portugal; 4 Instituto de Telecomunicações, Delegação da Covilhã, 1049-001 Lisbon, Portugal; 5 Research Center in Sports Performance, Recreation, Innovation and Technology (SPRINT), 4960-320 Melgaço, Portugal; 6 The Research Centre in Sports Sciences, Health Sciences and Human Development (CIDESD), 5001-801 Vila Real, Portugal; 7 Department of Exercise Physiology, Faculty of Educational Sciences and Psychology, University of Mohaghegh Ardabili, Ardabil, 56199-11367 Iran; 8 Faculty of Sport Sciences, University of Extremadura, 10003 Cáceres, Spain; 9 Department of Motor Performance, Faculty of Physical Education and Mountain Sports, Transilvania University of Braşov, 500068 Braşov, Romania

**Keywords:** Physiology, Health care

## Abstract

The evolution of female soccer is related to the increase in high-intensity actions and choosing the abilities that best characterize the players' performance. Determining the capabilities that best describe the players' performance becomes essential for coaches and technical staff to obtain the results more efficiently within the competitive calendar. Thus, the study aimed to analyze the correlations between performance in the 20-m sprint tests with and without the ball and the Zigzag 20-m change-of-direction (COD) test without the ball in professional female soccer players. Thirty-three high-level professional female soccer players performed the 20-m sprint tests without a ball, 20-m sprint tests with the ball, and the Zigzag 20-m COD test without the ball. The shortest time obtained in the three trials was used for each test. The fastest time in the three trials was used for each test to calculate the average test speed. The Pearson product–moment correlation test was applied to analyze the correlation between the performance in the tests. Pearson's product–moment correlation test was used to analyze the correlation between the performance in the trials, with a significance level of α < 0.05. The average speed in the 20-m sprint tests with ball showed very large and significant correlations with the speed in the Zigzag 20-m COD test (r = 0.822; p < 0.001; 95% CI = 0.666 to 0.909). The 20-m sprint tests with ball and 20-m sprint tests without ball showed moderate, positive and significant correlation (r = 0.363; p = 0.038; 95% CI = 0.023–0.628). The tests of 20-m sprint tests without ball and Zigzag 20-m COD test also showed moderate, positive and significant correlation (r = 0.415; p = 0.016; 95% CI = 0.084–0.664). The female–female soccer players with a better ability to change direction may also have a better technical ability to drive the ball at high speed. However, they will not necessarily be the fastest in the linear sprint without the ball. Coaches and technical staff may choose to perform tests seeking efficiency and practicality, especially in a congested competitive period.

## Introduction

Female soccer players have increased by around 30% since the 2000s^1^. The latest report presented by FIFA, referring to the 2019 FIFA World Cup, shows an evolution that female soccer has been going through, highlighting a substantial increase in high-intensity actions^2^. Thus, it is essential to investigate high-intensity activities in female soccer, as these actions can precede the goal and be fundamental to winning the game^3,4^.

To identify the high-intensity performance of female soccer players, sprints test in a straight line without possession of the ball and with the change of direction (COD) without possession of the ball are commonly used^5–10^. Conducting only these evaluations may not allow for identifying the technical quality of female soccer players, so the inclusion of ball handling in the evaluation assessments can add value to the performance analysis. Although the inclusion of the ball may increase the specificity of the soccer test, this may influence the relationships between test outcomes. For example, COD tests are typically moderately correlated with linear sprint since velocity is a determinant of COD^11^. However, the presence of the ball may vary the ability to move faster while holding the ball control, which interferes with the ultimate performance.

The selection of tests to evaluate the technical and physical performance of female soccer players technical and physical performance must consider the time available for carrying them out within the competitive calendar. Thus, studies that analyzed the relationships between assessments may justify the choice of tests more efficiently. A study on youth soccer players revealed correlations between the 10-m and 30-m sprint tests and the ZigZag COD test (r = 0.567 and r = 0.744, respectively)^12^. Another study of youth soccer players revealed correlations between the flying 20-m sprint test and the ZigZag COD test (r = 0.603)^13^. Also, a study on soccer players showed correlations between Zig-Zag with and without a ball^12^. The relationship between linear and change-of-direction has also been observed in studies conducted on female players^8,14^.

This information can help coaches and technical staff choose the tests. Still, to our knowledge, there is no analysis of correlations between the test trials with professional female soccer, and the investigation of correlation with the sprint with ball handling was not found. Therefore, the present study is necessary to investigate which characteristics are correlated between the tests and justify the choice of evaluations. This study will be one of the few to identify how sprint performance with and without the ball can be related to a change-of-direction test. Such analysis will allow understanding if the change-of-direction test can be highly correlated with one of the sprints (with and without the ball), offering an opportunity to possibly reduce the number of trials for application in practical scenarios.

Thus, because the evolution of female soccer is related to the increase in high-intensity actions, choosing the evaluations that best characterize the players' performance becomes essential for coaches and technical staff to obtain the results more efficiently within the competitive calendar. Therefore, the study aimed to analyze the correlations between performance in the 20-m sprint without a ball, 20-m sprint with a ball, and the Zigzag 20-m COD tests without a ball in professional female soccer players.

## Materials and methods

### Design and setting

This study followed a cross-sectional design. After one week before the end of the competitive season (August/2021), and after 1 day of the last training and 15 days of the last previous game, the players were assessed (in the following order) for three tests: (i) 20-m sprint tests without the ball; (ii) 20-m sprint tests with the ball; e (iii) Zigzag 20-m COD test. A standardized warm-up protocol consisting of 5 min of dynamic stretching followed by 5 min of actions that simulated the tests preceded the execution of tests. Three trials were performed for each test, with 5 min of rest. Between tests, an additional 5 min period was provided for rest. Hydration was not allowed during the period. The assessments were performed in the morning, starting at 9 am. The players performed the assessment evaluations in natural turf and environmental conditions of 20 °C. Players were asked to refrain from the consumption of consuming refreshing drinks and to maintain a regular diet before the assessments.

### Participants

The sample size was calculated a prior for before the study design. The sample calculation performed in the G * Power 3.1 program (Heinrich Heine, Düsseldorf, Germany) with the result of the correlation between the performance in the 30-m sprint tests without the ball and Zigzag 20-m COD test without the ball in soccer players (r = 0.744, p = 0.01) identified the need for at least 17 players for the study sample, taking into account power of 95%^12^. Thirty-three professional female soccer players (age: 23.48 ± 5.72 years; body mass: 60.73 ± 7.02 kg; height: 165.79 ± 6.87 cm) with at least 1 year of experience in professional competitions voluntarily participated in the study. The women's soccer players were part of two professional women's soccer teams in Brazil that went to the final stages of the Brazilian championship of the first division in; in addition, they were part of the cast of the player teams that were periodically summoned to the Brazilian women's soccer team. The team group trained 5 to 6 days a week, with an average training time of 1 h and 10 min.

Additionally, the team competed once or twice a week, specifically on weekends. The sample consisted of eight central defenders, four full-backs, ten midfielders, and eleven forwards. Players were informed of the risks and benefits of the assessment before data collection, and team officials provided written informed consent before the investigation. The eligibility criteria were: (i) the team medical department must clear the players, the team's medical department must clear the players; (ii) they have played professional games during the season, and (iii) only being field players. Goalkeepers were excluded from the sample due to the low specificity of the assessments performed for the position. The study was approved by the Institutional Review Board (or Ethics Committee) of the Polytechnic Institute of Viana do Castelo, School of Sport and Leisure (code: CTC-ESDL-CE001-2021). This study was conducted by the World Medical Association's code of ethics (Declaration of Helsinki), printed in the British Medical Journal (July 18, 1964).

### Procedures

**Figure 1:**
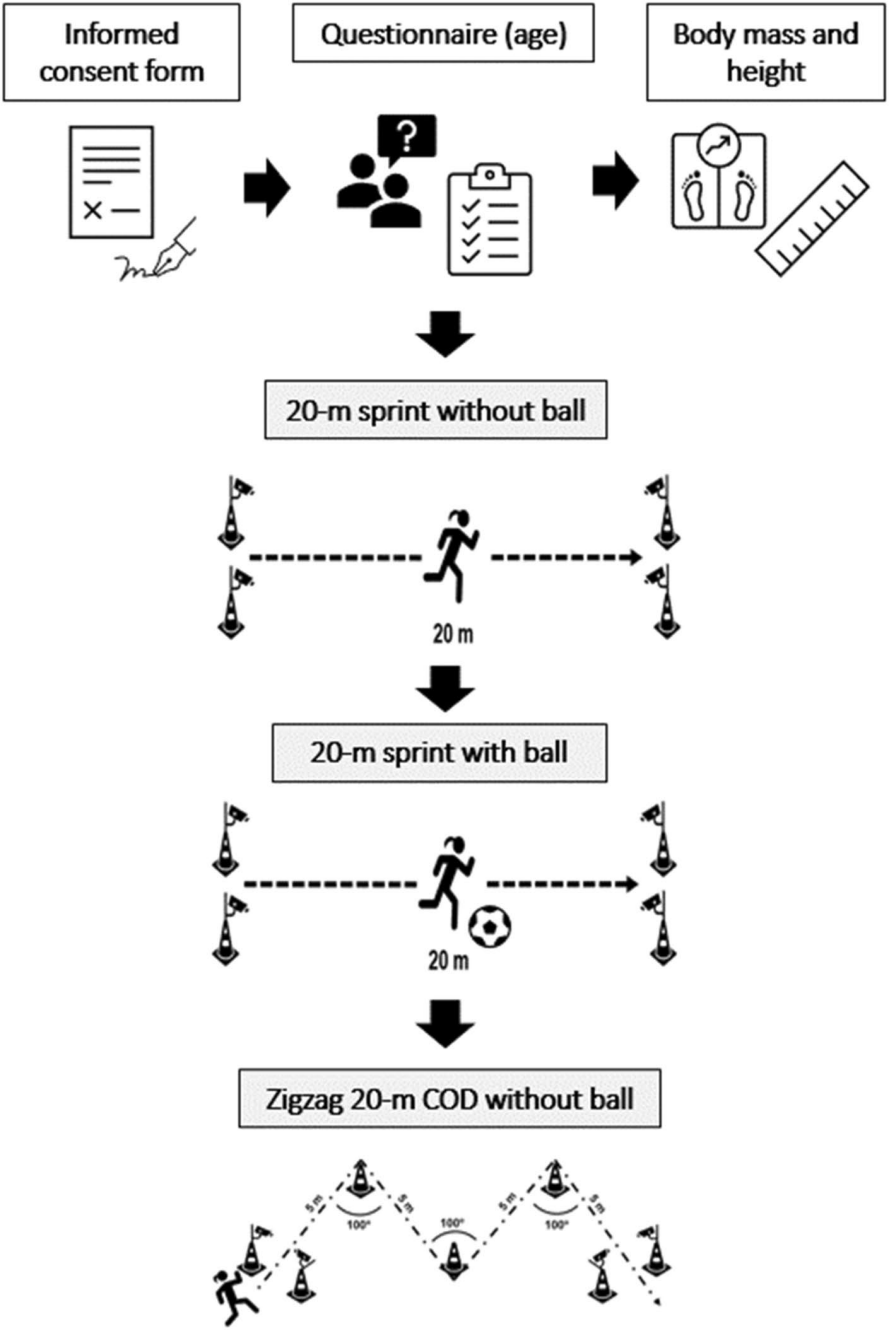
Data collection procedures. *COD* change-of-direction.

Anthropometric profile and physical performance assessments were performed in the same session one week after the end of the Brazilian championship at the team's training center, which was the preparatory period for state competition. Initially, the objectives and methodological procedures of the study were explained to the players, and the free and informed consent form was signed. The procedures performed are shown in Fig. 1.

#### Anthropometric profile

The evaluations took place at 9 am, being carried out 1 h after the first meal of the day and were carried out 1 h after the day's first meal. For body mass body assessment, the players were asked to be barefoot on a scale with a resolution of 100 g (G-TECH—Accumed Produtos Médico Hospitalares Ltda, Duque de Caxias, Brazil). To measure height, the players were asked to remain in an orthostatic position with their backs against the wall, where a measuring tape with a resolution of 1 mm was vertically fixed to the wall. With this, the evaluator measured the height of the players.

#### Physical performance profile

This was evaluated through three tests conducted in the following order: (I) 20-m sprint tests without a ball, (II) 20-m sprint tests with the ball, and (III) Zigzag 20-m COD test without a ball, all tests were evaluated using a triggered timing system by a photocell system positioned at the beginning and end of the course, with a resolution of 1 ms and dual-beam system (Speed Teste Fit—CEFISE, São Paulo, Brasil). The photocells were placed at the height of 100 cm from the ground. Three attempts were made in each test, each h an interval of 5 min.

#### 20-m sprint tests without the ball

The players were instructed to start with one foot positioned on a mark 30 cm from the first photocell 30 cm from the first photocell on a mark. Then, after the sound signal using of single, the player should run at maximum intensity until completely transposing the second photocell so that the timing system could capture the time it took the athlete to cover the distance of 20 m; the player should perform this test in the shortest possible time^11,15^. A cone was placed 5 m from the end point of the test so that they could run to it without slowing down before crossing the second photocell (Fig. 1). The shortest time in seconds measured in the three attempts was considered. The average speed on the route was calculated from the disc distance omittance covered in the test by dividing the running time and converted to kilometers per hour.

#### 20-m sprint tests with the ball

The players were instructed to start with one foot positioned on a mark 30 cm from the first photocell 30 cm from the first photocell on a mark. Then, after the sound signal using stimuli, the player should run at maximum intensity, driving the ball until completely transposing the second photocell so that the timing system could capture the time it took the player to cover the distance of 20 m, the player should perform this test in the shortest possible time. The players were instructed to drive the ball with the outside of the foot to maintain possession and control of the ball during the course; they were used to this type of driving during training and games. The players performed 5 to 8 touches on the ball during the test. The players performed 5 to 8 contacts on the ball during the test to complete the course. A cone was placed five meters from the trial's endpoint so that they could run to it without slowing down before crossing the second photocell (Fig. 1). The shortest time measured in seconds in the three attempts was considered. The average speed on the route was calculated from the distance from a distance covered in the test by dividing the running time and converted to kilometers per hour.

#### Zigzag 20-m COD test without ball

The players were instructed to start with one foot positioned on a mark 30 cm from the first photocell 30 cm from the first photocell on a mark. Then, after the sound signal using a whistle, the player should run at maximum intensity until completely transposing the second photocell so that the timing system can capture the time it took the player to cover the distance of 20 m (4 sections of 5 m marked with cones fixed at 100° angles, always performing the displacements on the outside of the cones), they should perform conduct this test in the shortest possible time^11,15^. A cone was placed five meters from the trial's endpoint so that they could run to it without slowing down before crossing the second photocell (Fig. 1). The shortest time in seconds measured in the three attempts was considered. The average speed on the route was calculated from a distance covered in the test by dividing the running time and converted to kilometers per hour.

### Statistical analysis

The variables are presented through descriptive analysis using mean, standard deviation, and 95% confidence interval (CI). The average coefficient of variation between trials in the tests was calculated. Data from 20-m sprint tests without the ball (p = 0.153), 20-m sprint tests with the ball (p = 0.453), and the Zigzag 20-m COD test (p = 0.254) were normally distributed according to the Shapiro–Wilk test. Pearson's product–moment correlation coefficient was used to determine the relationship between the 20-m sprint tests without a ball, the 20-m sprint tests with the ball, and the Zigzag 20-m COD test. As a complementary qualitative correlation assessment, r equal to 0 was considered trivial, between 0.1 and 0.3 as small, 0.3 and 0.5 as moderate, 0.5 and 0.7 as extensive, 0.7 and 0.9 as very large, 0.9 and 1 as nearly perfect, and 1.0 as perfect^16^. Intraclass correlation coefficients 2. k and Cronbach's alpha reliability coefficients were used to determine the players' intertrial reliability on physical performance tests. Data analysis was performed using SPSS 21.0 software (IMB, Chicago, IL). The significance level adopted was α < 0.05. The statistical power of the correlations was further calculated using the program G * Power 3.1 (Heinrich Heine, Düsseldorf, Germany).

### Ethics approval and consent to participate

All participants were familiarized with the training protocols. All players with parents signed informed consent before the investigation. The study was conducted according to the guidelines of the Declaration of Helsinki. The study was approved by the Institutional Review Board (or Ethics Committee) of the Polytechnic Institute of Viana do Castelo, School of Sport and Leisure (code: CTC-ESDL-CE001-2021).

### Consent for publication

Written informed consent has been obtained from the subject (s) to publish this paper.

## Results

Description of the performance obtained in the 20-m sprint tests without a ball, 20-m sprint tests with the ball, and Zigzag 20-m COD tests are shown in Table 1. The female soccer players presented an average coefficient of variation between attempts of 1.51 ± 0.95%, the intraclass correlation coefficient of 0.913, and Cronbach'’s α coefficient of 0.910 in the 20-m sprint tests without the ball. For the 20-m sprint tests with the ball, the average coefficient of variation between attempts was 3.03 ± 2.24%, the intraclass correlation coefficient of 0.779, and Cronbach's α coefficient of 0.773. The Zigzag 20-m COD test speed without a ball presented an average coefficient of variation between trials of 1.58 ± 1.03%, the intraclass correlation coefficient of 0.862, and Cronbach'’s α coefficient of 0.870.

**Table 1:** Description of the performance obtained in the 20-m sprint tests without the ball, 20-m sprint tests with the ball, and Zigzag 20-m COD test.

Tests	Mean	SD	95% CI	
			Lower	Upper
20-m sprint without ball (km/h)	22.39	0.80	22.10	22.67
20-m sprint with ball (km/h)	20.37	1.11	19.97	20.76
Zigzag 20-m COD (km/h)	13.81	0.54	13.62	14.00

*SD* standard deviation, *CI* confidence interval, *COD* change-of-direction.

**Figure 2:**
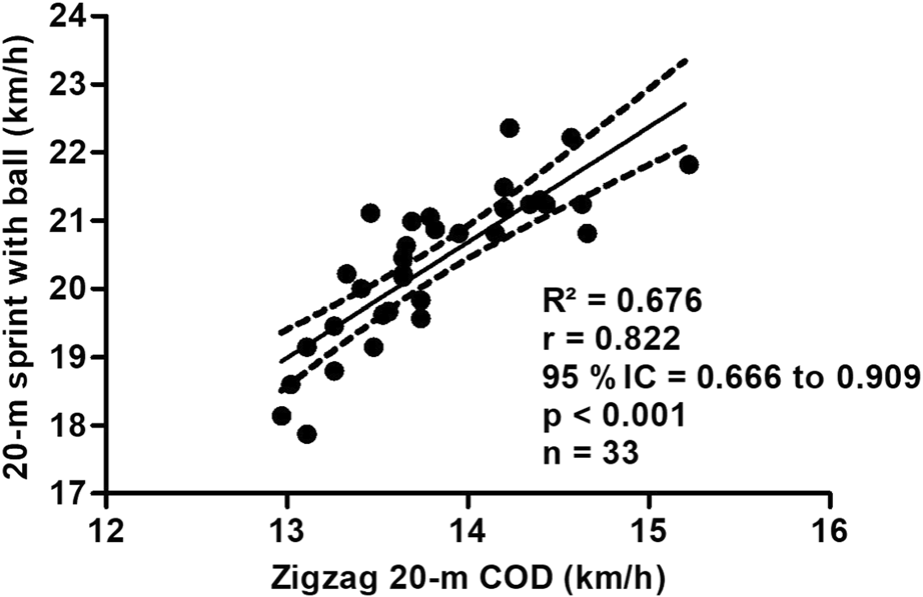
Substantial huge, positive, significant correlation between performance in the 20-m sprint tests with the ball and the Zigzag 20-m change-of-direction (COD) test of professional female soccer players.

Figure 2 shows the correlation between the performance in the 20-m sprint tests with the ball and the Zigzag 20-m COD test of professional soccer players. The performance in the 20-m sprint tests with ball showed a significant correlation with the performance in the Zigzag 20-m COD test, this correlation being very large and positive (R^2^ = 0.676; r = 0.822; p < 0.001; 95% CI = 0.666–0.909; power = 0.999).

**Figure 3:**
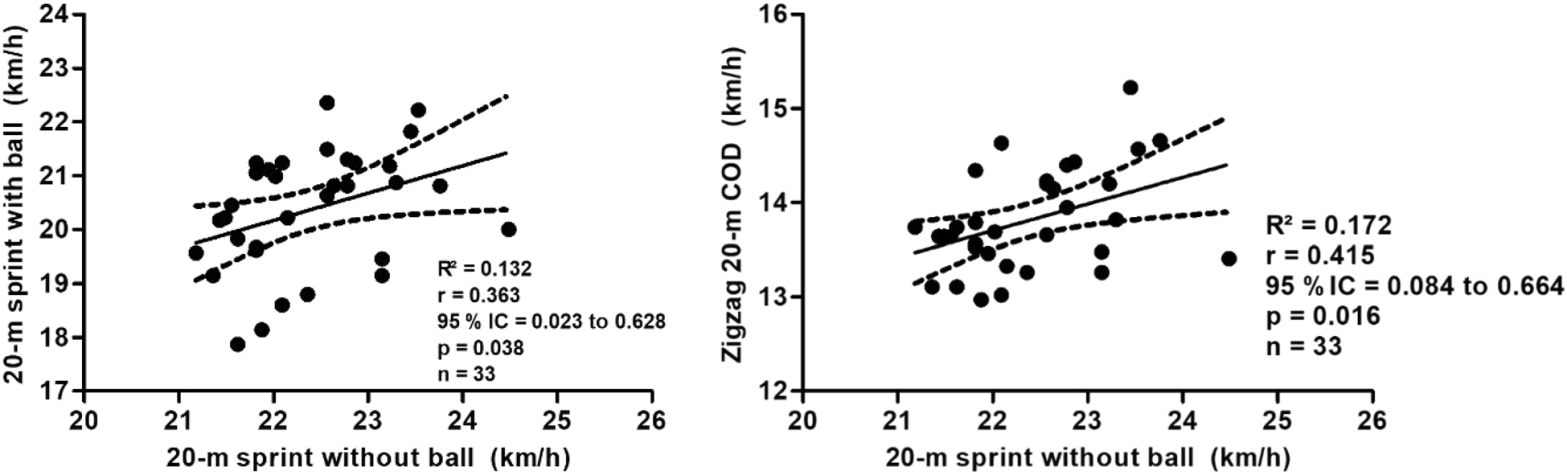
Moderate, positive, and significant correlation between the 20-m sprint tests with the ball and the 20-m sprint tests without the ball. Moderate, positive, and significant correlation between the 20-m sprint tests without the ball and the Zigzag 20-m change-of-direction (COD) test.

In Fig. 3, the other results of the correlation tests between the tests are presented. The 20-m sprint tests with ball and 20-m sprint tests without ball showed moderate, positive and significant correlation (R^2^ = 0.132; r = 0.363; p = 0.038; 95% CI = 0.023–0.628; power = 0.561). The tests of 20-m sprint tests without ball and Zigzag 20-m COD test also showed moderate, positive and significant correlation (R^2^ = 0.172; r = 0.415; p = 0.016; 95% CI = 0.084–0.664; power = 0.690).

## Discussion

The current cross-sectional study analyzed the relationships between 20-m sprint tests with and without ball control and a Zigzag 20-m COD test without a ball performed by female soccer players. The primary evidence is that the 20-m sprint most testate ball largely correlated with The Zigzag COD test. At the same time, only moderate correlations were found between the 20-m sprint test without the ball and the 20-m sprint test with the ball largely correlated with the Zigzag COD test.

The current research revealed that the Zigzag COD test had moderate relationships with the 20-m sprint test without the ball. The values obtained (r = 0.415; p = 0.016; 95% CI = 0.084–0.664) in our research were similar to a previous study that revealed correlations (r = 0.567) 10-m sprint test with ZigZag COD test in youth soccer players^12^ and one study that also revealed showed large significant correlations (r = 0.603) between 20-m sprint test and ZigZag COD test in youth soccer players^13^. The Zig Zag COD test provides four sets of 5-m linear running, with three COD at 100°. Although this test allows tremendous efficiency in accelerating and decelerating over very short distances, the performance in the trial was moderately correlated with the linear sprint test without the ball. The correlation can be justified by the fact that the COD deficit (difference between linear sprint and sprint with the change of direction) can be more related to faster power than the COD time. COD time is highly associated with linear sprint. On the other hand, COD deficit is related to the skill of changing direction, which means that it is a more specialized motor skill. This can be observed in a study testing relationship between speed and power and COD performance at the ZigZag COD test^11^. How players approach the COD point (braking and acceleration phases) or the specialization level for taking advantage of COD maneuvers can justify that sprint without a ball is not correlated mainly with the ZigZag test^17^.

Interestingly, the 20-m sprint with the ball was mainly correlated with the ZigZag COD test, which was not observed for sprinting without the ball. Previous studies compared performances in running flying and COD tests with and without a ball, revealing that the presence of a ball is detrimental to time performance compared to test trials without a ball^18,19^. Possibly, the locomotive task of dribbling may produce changes in motor control constrained by restricted peripheral vision due to watching the ball's position and adjusting the movement to the ball positioning^20^. Thus, the ball condition requires an adaptation on locomotor profile, providing greater participation of coordinative skills that partially may align with COD. Although COD requires a well-applied technique related to foot placement and posture, it is also possible that specific-related drill-based process can participate in the explanation of acting better in changes of direction. However, this possibility deserves further research and cannot be confirmed in the current literature.

This study had some limitation limitations. The convenience sampling and the small number of participants constrain the possible generalization of the findings. This is one of the reasons that does not allow splitting the analysis by playing positions. Having a small sample and being highly contextual does not allow us to suggest the evidence as a generalization; for that reason, any conclusion must be limited to the current context. Moreover, the ZigZag COD test was not performed with the ball, which would be essential to have some comparisons with sprint with the ball. Finally, relationships with other physical qualities and technical skills were not conducted, which should be considered in future studies. Finally, biomechanical analysis using motion cameras can provide a step forward in understanding the player’s patterns and eventually improve the possibilities of comparing the process conducted to the evidence found in our study^21,22^.

Although the study's limitations, the current research results are interesting for the scientific community since it is the first, as far we know, that found substantial correlations between linear sprinting with a ball and ZigZag COD performance without the ball. Thus, coaches and technical staff may choose either 20-m sprint tests with a ball or a Zigzag 20-m COD test without a ball seeking better efficiency and practicality, especially in a congested competitive period. Future research should consider replicating the design in a larger sample and identify if technical ability may mediate the final results while adding 3D analysis for comparing the biomechanics of the players. Future research should consider replicating the design in a larger sample and identify if technical ability may mediate the final results.

## Conclusions

The female–female soccer players with a better ability to change direction may also have a better technical ability to drive the ball at high speed. However, they will not necessarily be the fastest in the linear sprint without the ball. However, the small sample tested does not allow us to generalize the evidence. We propose that physical tests may include both tests with and without balls in linear and non-linear trajectories since they will discriminate better the player's performance.

## Supplementary Information

Supplementary Information.

## Data Availability

The data presented in this study are available as a supplementary file.

## Abbreviations

FIFA: Fédération Internationale de Football Association
COD: Change-of-direction
